# Efficacy of the combination of umbilical fat and venous blood on limb salvage

**DOI:** 10.3389/fbioe.2026.1775975

**Published:** 2026-02-25

**Authors:** Erhan Hafiz

**Affiliations:** Gaziantep University Faculty of Medicine Department of Cardiovascular Surgery, Gaziantep, Türkiye

**Keywords:** autologous conditioned plasma, chronic limb-threatening ischemia, limb salvage, stromal vascular fraction, umblical fat

## Abstract

**Objective:**

To evaluate the efficacy of combined umbilical adipose tissue and venous blood–derived stromal vascular fraction (SVF) on limb perfusion, pain and functional recovery in patients with chronic limb-threatening ischemia (CLTI).

**Methods:**

A total of 20 patients aged between 36 and 82 years (mean age: 60.3 ± 11.2 years) were included in the study. Ankle-Brachial Index (ABI), pain scores and pain-free walking distance were evaluated before treatment and at follow-up at 3, 6, 9 and 12 months.

**Results:**

At 12 months, ABI increased significantly, Visual Analog Scale (VAS) decreased, while pain-free walking distance improved at 9 and 12 months compared to baseline. Left ventricular ejection fraction (LVEF) showed a non-significant rise (54.6%–55.2%, p = 0.072), while renal function improved modestly (creatinine 1.12 → 1.09 mg/dL, eGFR 78 → 80.5 mL/min/1.73 m^2^, p = 0.034). Angiography revealed enhanced vessel visualization and collateral formation.

**Conclusions:**

Combined Autologous conditioned plasma (ACP) and adipose-derived SVF injections effectively improved limb perfusion, pain, lesion severity and functional outcomes in CLTI patients, suggesting a promising therapeutic approach for peripheral arterial disease (PAD).

## Introduction

Chronic limb-threatening ischemia (CLTI) represents the advanced stage of peripheral arterial disease (PAD), characterized by multilevel arterial occlusions and insufficient blood flow to sustain tissue viability. Clinically, it presents with rest pain, non-healing ulcers, or gangrene and affects <20% of PAD patients, corresponding to approximately 1.28% of adults over 40 years, with nearly two million cases annually in the United States alone (Eid et al., 2021; Bekwelem and Hirsch, 2017; Beckman et al., 2021). If untreated, CLTI often progresses to amputation, leading to functional decline, reduced quality of life and high healthcare costs (Teraa et al., 2016). Despite advancements in revascularization techniques, amputation rates remain high, with 1-year mortality exceeding 40% in many cases (Teraa et al., 2016). Certain populations, including rural, socioeconomically disadvantaged and racial or ethnic minority groups, are disproportionately affected (Minc et al., 2021).

Traditional management relies on endovascular therapy and surgical bypass; however, innovative approaches such as autologous stem cell transplantation (ASCT) have emerged (Levin et al., 2020). ASCT has demonstrated clinical benefits across multiple diseases, including hematologic malignancies, autoimmune conditions and metabolic disorders, while also showing promise in the treatment of CLTI (Kamber et al., 2015; Cook et al., 2011; Cook et al., 2016; Lepik et al., 2019; Wang et al., 2015; Zahid et al., 2017; Li et al., 2014; Xie et al., 2018; Bura et al., 2014).

Adipose-derived stem cells (ADSCs) are of particular interest due to their abundance, accessibility, differentiation potential and reparative effects, including promotion of angiogenesis and modulation of inflammation (Zhang et al., 2020). Venous blood provides an additional source of hematopoietic and immune cells with regenerative potential and its minimally invasive collection enhances clinical applicability (Zhao et al., 2017; Lawall et al., 2010).

This study investigates the therapeutic potential of combining adipose tissue-derived stem cells with venous blood-derived components for patients with CLTI, aiming to assess their synergistic effects on tissue healing and limb salvage.

## Methods

This prospective interventional case series evaluated the efficacy of combined adipose tissue and venous blood in patients with critical limb-threatening peripheral artery disease (PAD) who lacked revascularization options. Twenty patients were enrolled after providing written informed consent.

### Patient selection

Twenty patients aged ≥18 years with CLTI were enrolled in accordance with the Global Vascular Guidelines. Inclusion criteria comprised an ABI ≤0.4 or transcutaneous oxygen pressure (TcPO_2_) ≤30 mmHg, accompanied by non-healing ischemic ulcers or gangrene. Patients with multilevel tibial occlusions confirmed by angiography and deemed unsuitable for surgical or endovascular revascularization were eligible. Exclusion criteria included systemic infection, severe cardiac dysfunction (EF <30%), dialysis-dependent renal failure, malignancy, coagulation disorders and ongoing immunosuppressive therapy. A pragmatic sample size of 20 patients, consistent with prior pilot studies on cell-based therapies, was chosen to evaluate safety and feasibility rather than statistical efficacy.

### Inclusion criteria

Patients aged ≥18 years were included if they had CLTI, defined by an ABI ≤0.4 and/or non-healing ischemic ulcers or gangrene. Angiographic evidence of multilevel occlusions or severe stenosis in at least two major lower limb arteries (anterior tibial, posterior tibial, or peroneal) was required. Patients were eligible when revascularization via endovascular or surgical approaches was deemed unfeasible. This population typically presents with chronic ulcers, infection, or gangrene, often necessitating major limb amputation in the absence of alternative therapies, underscoring the need for innovative interventions.

### Intervention and preparation ACP–SVF preparation

Autologous stromal vascular fraction (SVF) was prepared from abdominal subcutaneous adipose tissue combined with autologous conditioned plasma (ACP) obtained from peripheral venous blood, using ARTHREX ACP and fat transfer kits.

### Venous blood collection and ACP preparation

Autologous conditioned plasma (ACP) was prepared from peripheral venous blood using a standardized protocol. A total of 30 mL of venous blood was obtained from each patient under sterile conditions, with 15 mL collected into each ACP syringe (Arthrex ACP® Double Syringe System, Arthrex Inc., Naples, FL, United States of America). Sodium citrate (1 mL) was added to each syringe as an anticoagulant to prevent premature coagulation. The samples were then centrifuged according to the manufacturer’s protocol using a dedicated centrifuge at 1,500 rpm for 5 min at room temperature. Following centrifugation, the plasma fraction enriched with platelets and plasma-derived bioactive factors was separated from the erythrocyte layer using the built-in double-syringe system and collected via a three-way stopcock. The resulting ACP was visually inspected to ensure absence of red blood cell contamination. The ACP obtained from both syringes was pooled and maintained under sterile conditions until immediate combination with the stromal vascular fraction (SVF) for intramuscular administration. No exogenous activation agents were added and ACP was used fresh in all patients.

### Adipose tissue harvesting and stromal vascular fraction (SVF) isolation

Stromal vascular fraction (SVF) was obtained from autologous abdominal subcutaneous adipose tissue using a standardized mechanical processing protocol. All procedures were performed under sterile conditions. The periumbilical region was disinfected and local anesthesia was administered approximately 2 cm below the umbilicus. A tumescent solution consisting of 0.5 mg adrenaline, 10 mL bupivacaine and 100 mL normal saline was infiltrated into the subcutaneous tissue. After a waiting period of approximately 10 min, adipose tissue was aspirated using gentle negative pressure through a blunt cannula to minimize cellular damage. A total volume of 30 mL of adipose tissue was collected from each patient. The harvested adipose tissue was processed mechanically without enzymatic digestion. Mechanical emulsification was performed by sequential passage of the lipoaspirate through cannulas of decreasing diameters (2.4 mm, 1.4 mm and 1.2 mm) to disrupt adipose clusters and release the stromal vascular fraction. The emulsified fat was then centrifuged according to the manufacturer’s protocol (Program B). Following centrifugation, the upper oil (triglyceride) layer was discarded and the intermediate aqueous layer containing cellular components was collected. The material was subsequently filtered and centrifuged a second time to further purify the SVF.

The final SVF pellet was resuspended and combined with ACP to obtain a homogeneous injectable suspension. The preparation was used immediately for intramuscular administration without further manipulation or cryopreservation.

### Final preparation and application

The final SVF suspension was combined with ACP to obtain a homogeneous injectable product. The prepared solution was administered as a single-session treatment via multiple intramuscular injections into the ischemic lower limb. Injections were performed under sterile conditions and targeted the major muscle groups of the affected limb, including the gastrocnemius, soleus, tibialis anterior and peroneal muscles, depending on the anatomical distribution of ischemia. The injection sites were distributed along the calf and distal lower limb, with particular emphasis on regions adjacent to ischemic ulcers or areas supplied by occluded tibial vessels. The total injection volume was evenly divided among multiple injection points to ensure homogeneous local distribution and to enhance cell retention within ischemic tissues. No additional imaging guidance was used during administration. All patients received a single combined SVF–ACP administration and no repeat dosing was performed during the follow-up period.

Because the SVF–ACP product was prepared as a point-of-care formulation for immediate administration, no formal product characterization (total nucleated cell yield, viability testing, or flow-cytometric immunophenotyping) was performed. Concomitant antiplatelet and anticoagulant therapies were maintained unchanged in all patients according to standard clinical practice for chronic limb-threatening ischemia. No temporary suspension or modification of antiaggregation or anticoagulation therapy was performed prior to the procedure in order to avoid thrombotic risk in this high-risk population. Patients were not stratified according to antiplatelet regimen.

### Data collection and evaluation

#### Collecting demographic and clinical data

Each patient’s background, including demographic details, medical history and condition prior to treatment, was thoroughly documented. Following the procedure, regular clinical follow-ups were conducted to track progress and gather essential data for evaluation.

#### Assessing functional improvements assessment of functional recovery

Identification of critical limb-compromised PAD, in which blood flow to the lower extremities is severely reduced, was primarily defined by the ABI. An ABI of 0.4 or less indicated severe PAD, while non-healing ulcers reflected inadequate tissue perfusion.

Pain levels were assessed using the Visual Analog Scale (VAS), allowing comparison of pain intensity before and after treatment. Pain-free walking distance was measured using a six-minute walking test, reflecting changes in mobility and quality of life.

#### Cardiac and renal function monitoring

Cardiac and renal functions were systematically assessed at baseline and at 3, 6, 9 and 12 months post-intervention. Cardiac evaluation included echocardiographic measurement of left ventricular ejection fraction (LVEF), resting heart rate monitoring and electrocardiography (ECG). Renal assessment comprised serum creatinine, estimated glomerular filtration rate (eGFR) and blood urea nitrogen (BUN). All values were compared with baseline to evaluate intervention-related changes.

### Statistical analyses

Data were analyzed using SPSS version 21.0. Descriptive statistics, including mean, standard deviation and percentages, were calculated. Normality was assessed using the Kolmogorov–Smirnov and Shapiro–Wilk tests. Paired-samples t-tests were used to compare continuous variables before and after the procedure, while chi-square tests evaluated categorical variables. A p-value <0.05 was considered statistically significant.

## Results

A total of 20 patients were enrolled, including 13 men (65%) and seven women (35%), with a mean age of 58.6 ± 12.3 years. Eight patients (40%) were smokers. Comorbidities included diabetes mellitus (DM) in 14 patients (70%), hyperlipidemia (HPL) in 10 (50%), hypertension (HT) in 6 (30%), coronary artery disease (CAD) in 6 (30%) and chronic renal failure (CRF) in 2 (10%) (Table 1).

**TABLE 1:** Characteristics of the patients.

Age (Min.-Max/Ort. ± ss)	35–85/58.61 ± 12.33
Gender – n (%)	
Female	7 (35)
Male	13 (65)
Smoking-n (%)	
No	12 (60)
Yes	8 (40)
HPL-n (%)	
No	10 (50)
Yes	10 (50)
DM-n (%)	
No	6 (30)
Yes	14 (70)
HT-n (%)	
No	14 (70)
Yes	6 (30)
CRF-n (%)	
No	18 (90)
Yes	2 (10)
CAD-n (%)	
No	18 (90)
Yes	2 (10)

Min, Minimum; Max, Maximum; HPL, hyperlipidemia; DM, diabetes mellitus; HT, hypertension; CRF, chronic renal failure; CAD, coronary artery disease.

When patients’ ABI, VAS and pain-free walking values before and after surgery were examined, the ABI, value at 12 months postoperatively was found to have increased significantly compared with preoperative values, while the VAS, score decreased significantly.

In addition, pain-free walking distance at 9 and 12 months postoperatively increased significantly compared with both preoperative values and those measured at 3 months post-surgery (Table 2).

**TABLE 2:** Comparison of ABI, VAS and pain free walking distance.

Time	ABI	VAS	Pain free walking distance (m)
Preop	0.40 ± 0.05^a^	6.32 ± 0.95^a^	35.0 ± 12.11^a^
3rd month	0.43 ± 0.06	6.29 ± 0.90	38.0 ± 9.08^b^
6th month	0.45 ± 0.06	6.15 ± 0.83	43.2 ± 5.45
9th month	0.47 ± 0.07	5.89 ± 0.87	50.25 ± 4.76
12th month	0.50 ± 0.06^a^	5.21 ± 0.46^a^	55.12 ± 4.47^a,b^
p	0.032	0.048	0.036

The letters “a” and “b” denote statistically significant differences. Groups with the same letter differ significantly from each other. “Preop” refers to the preoperative period, while “3rd month,” “6th month,” “9th month,” and “12th month” indicate 3, 6, 9, and 12 months after the operation, respectively.

Systemic effects were less pronounced. Left ventricular ejection fraction (LVEF) increased slightly from 54.6% to 55.2% at 12 months, without reaching statistical significance (p = 0.072). Serum creatinine decreased marginally from 1.12 to 1.09 mg/dL, while the estimated glomerular filtration rate (eGFR) increased to 80.5 mL/min/1.73 m^2^ at 12 months (p = 0.034). Blood urea nitrogen (BUN) levels demonstrated only minimal change, decreasing from 17.3 to 17.0 mg/dL (Table 3).

**TABLE 3:** Comparison of cardiac and renal parameters.

Time	LVEF (%)	Creatinine (mg/dL)	eGFR (mL/min/1.73 m^2^)	BUN (mg/dL)
Preop	54.60 ± 6.3	1.12 ± 0.23	78.5 ± 12.4^a^	17.3 ± 3.8^a^
3rd month	54.70 ± 6.1	1.11 ± 0.21	78.8 ± 12.0	17.3 ± 3.6^b^
6th month	54.90 ± 6.0	1.11 ± 0.20	79.2 ± 11.8	17.2 ± 3.5
9th month	55.10 ± 5.9	1.10 ± 0.20	79.8 ± 11.7	17.1 ± 3.5
12th month	55.20 ± 5.7	1.09 ± 0.19	80.5 ± 11.5^a^	17.0 ± 3.3^a,b^
p	0.072	0.074	0.034	0.036

The letters “a” and “b” denote statistically significant differences. Groups with the same letter differ significantly from each other. “Preop” refers to the preoperative period, while “3rd month,” “6th month,” “9th month,” and “12th month” indicate 3, 6, 9, and 12 months after the operation, respectively.

**FIGURE 1:**
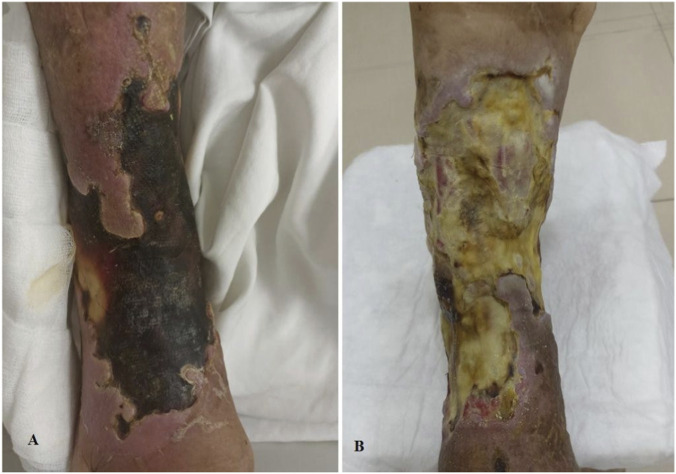
**(A)** Preoperative and **(B)** Postoperative limb image 3 months later.

Angiographic evaluation at 3 months demonstrated improved vessel visualization and collateral circulation (Figure 1), supporting the potential of adipose tissue- and venous blood–derived stromal vascular fraction to enhance revascularization in critical limb ischemia.

During the 12-month follow-up period, one patient required a minor amputation involving two toes, while no major limb amputations were performed in the study cohort.

## Discussion

This study presents a novel approach combining adipose-derived stem cells (ASCs) from periumbilical fat with autologous venous blood products for the treatment of CLTI. Unlike platelet-rich plasma (PRP) or bone marrow–derived mononuclear cell protocols, this method leverages both the regenerative capacity of ASCs and the trophic support of venous blood–derived factors. Our findings suggest improvements in vascular perfusion, lesion healing, functional outcomes and selected systemic parameters, highlighting the therapeutic potential of this strategy in ischemic vascular disease.

The significant increase in ABI values indicates enhanced microvascular perfusion and arterial remodeling. These effects are consistent with prior evidence demonstrating that ASCs secrete angiogenic factors such as VEGF, FGF-2, PDGF and HGF, thereby promoting endothelial proliferation and vessel formation (Lawall et al., 2010; Zhuang et al., 2019; Lozano et al., 2022). Venous blood products likely contributed to vessel stabilization through mediators such as PDGF-BB and TGF-β1. Variability in patient responses may reflect differences in diabetes duration, metabolic control and chronic inflammation, all of which influence angiogenic capacity (Yousefifard et al., 2020).

Symptomatic improvements, including reduced ischemic pain and increased pain-free walking distance, suggest benefits extending beyond hemodynamic changes. ASCs exert immunomodulatory effects by downregulating pro-inflammatory cytokines and enhancing anti-inflammatory mediators, while venous blood products may further support endothelial function and limit local inflammation.

Although changes in LVEF were modest and did not reach statistical significance, renal function demonstrated favorable trends, including improved eGFR and reduced creatinine levels. These findings are consistent with experimental data showing mesenchymal stem cell–mediated protection against ischemia-induced nephropathy (Fisher et al., 2015; Kukharchuk et al., 2024). Mechanistically, the observed effects likely arise from paracrine signaling, immunomodulation, extracellular vesicle–mediated transfer of regulatory molecules and modulation of the TGF-β pathway (Conte et al., 2019).

Recent advances in regenerative therapies for chronic limb-threatening ischemia have highlighted the importance of rigorous cellular characterization and controlled study designs. In this context, Kukharchuk et al. (2024) reported a comprehensive preclinical and randomized clinical investigation evaluating fetal progenitor cells (FPCs) for the treatment of chronic limb ischemia (Kukharchuk et al., 2024). Their study demonstrated significant improvements in wound healing, pain reduction, ankle–brachial index and prevention of major amputations, supported by detailed cellular phenotyping and long-term follow-up (Kukharchuk et al., 2024). Compared with this methodologically robust approach, the present study represents an early-stage, pragmatic clinical experience using mechanically processed autologous stromal vascular fraction combined with autologous conditioned plasma as a point-of-care therapy. While our approach lacks extensive cellular characterization and randomization, it offers a feasible and ethically accessible strategy for no-option chronic limb-threatening ischemia patients. Together, these studies illustrate complementary pathways in regenerative angiogenesis, ranging from highly standardized cell products to real-world autologous formulations that may inform future controlled trials.

Limitations of this study include the small sample size, lack of randomization and use of a single-dose protocol. Future studies should investigate repeated dosing strategies, optimized delivery routes, standardized characterization of ASCs and venous blood components and the incorporation of biomaterial carriers to enhance cell retention. Long-term follow-up assessing amputation-free survival, ulcer healing and recurrent ischemia is necessary to confirm the durability and clinical applicability of this approach.

An additional limitation is the lack of SVF product characterization (total viable cell yield, viability and immunophenotyping such as CD34^+^ progenitor frequency and mesenchymal/hematopoietic subsets), which limits biological standardization and cross-study comparability. In addition, the potential influence of ongoing antiplatelet or anticoagulant therapy on the biological activity of autologous conditioned plasma was not specifically evaluated, which represents an additional limitation of the present exploratory study. Future studies should incorporate standardized cell quantification and flow-cytometric profiling to improve reproducibility.

Amputation-related outcomes were not predefined as primary endpoints in this exploratory case series. Although no major amputations occurred, one patient required a minor toe amputation during follow-up, underscoring the need for larger, controlled studies evaluating amputation-free survival as a primary outcome.

Although the administration protocol was standardized within this study, further multicenter studies are required to optimize injection sites, dosing strategies and repetition intervals to establish a universally applicable protocol.

## Conclusion

In conclusion, the combined use of adipose-derived stem cells and autologous venous blood products represents a promising and biologically rational therapeutic strategy for patients with critical limb ischemia. By synergistically enhancing angiogenesis, modulating inflammation and supporting tissue repair, this approach demonstrated improvements in perfusion, functional outcomes and selected systemic parameters. Although preliminary, these findings provide a strong rationale for larger, randomized and longitudinal studies to further define the clinical efficacy, optimal treatment protocols and long-term benefits of this regenerative modality in ischemic vascular disease.

## Data Availability

The raw data supporting the conclusions of this article will be made available by the authors, without undue reservation.
